# Proceedings of the American Society of Microscopists

**Published:** 1887-11

**Authors:** 


**Proceedings of the American Society of Microscopists.** Ninth annual meeting, held at Chautauqua, N. Y., August 10–13, 1886.

The annual volume of proceedings of this society have in the past contained many valuable articles, and the present issue is fully equal to its predecessors. We are glad to note that Buffalo is so numerously and ably represented. Profs. Geo. E. Fell, M. D., and D. S. Kellicott, Dr. Geo. W. Lewis, and Messrs. Ward and Henry Mills, contribute excellent papers. A most admirable paper, by Hamilton Smith, LL.D., entitled, "A Contribution and the Life History of the Diatomatacea," is illustrated by a series of beautifully-colored plates.

